# Inequalities in reproductive, maternal, newborn and child health in Vietnam: a retrospective study of survey data for 1997–2006

**DOI:** 10.1186/1472-6963-12-456

**Published:** 2012-12-13

**Authors:** Henrik Axelson, Ulf-G Gerdtham, Björn Ekman, Dinh Thi Phuong Hoa, Tobias Alfvén

**Affiliations:** 1 The Partnership for Maternal, Newborn & Child Health (PMNCH), hosted by the World Health Organization, Avenue Appia 20, Geneva 27, 1211, Switzerland; 2 Department of Clinical Sciences, Malmö University Hospital, Lund University, Lund, Sweden; 3 Department of Economics, Lund University, Lund, Sweden; 4 Health Economics & Management, Institute of Economic Research, Lund University, Lund, Sweden; 5 Center for Primary Health Care Research, Lund University/Region Skåne, Lund, Sweden; 6 Social Medicine and Global Health, Department of Clinical Sciences, Malmö, Lund University, Malmö, Sweden; 7 Maternal and Child Health Department, Ministry of Health, Hanoi, Vietnam; 8 Hanoi School of Public Health, Hanoi, Vietnam; 9 Division of Global Health/IHCAR, Department of Public Health Science, Karolinska Institutet, Stockholm, Sweden; 10 Department of Paediatrics, Sachs’ Children’ Hospital, Södersjukhuset, Stockholm, Sweden

**Keywords:** Equity, Health care utilization, Health inequalities, Health outcomes, Reproductive health, Maternal health, Newborn health, Child health

## Abstract

**Background:**

Vietnam has achieved considerable success in economic development, poverty reduction, and health over a relatively short period of time. However, there is concern that inequalities in health outcomes and intervention coverage are widening. This study explores if inequalities in reproductive, maternal, newborn and child health and nutrition changed over time in Vietnam in 1997–2006, and if inequalities were different depending on the type of stratifying variable used to measure inequalities and on the type of outcome studied.

**Methods:**

Using data from four nationally representative household surveys conducted in 1997–2006, we study inequalities in reproductive, maternal, newborn and child health and nutrition outcomes and intervention coverage by computing concentration indices by living standards, maternal education, ethnicity, region, urban/rural residence, and sex of child.

**Results:**

Inequalities in maternal, newborn and child health persisted in 1997–2006. Inequalities were largest by living standards, but not trivial by the other stratifying variables. Inequalities in health outcomes generally increased over time, while inequalities in intervention coverage generally declined. The most equitably distributed interventions were family planning, exclusive breastfeeding, and immunizations. The most inequitably distributed interventions were those requiring multiple service contacts, such as four or more antenatal care visits, and those requiring significant support from the health system, such as skilled birth attendance.

**Conclusions:**

Three main policy implications emerge. First, persistent inequalities suggest the need to address financial and other access barriers, for example by subsidizing health care for the poor and ethnic minorities and by support from other sectors, for example in strengthening transportation networks. This should be complemented by careful monitoring and evaluation of current program design and implementation to ensure effective and efficient use of resources. Second, greater inequalities for interventions that require multiple service contacts imply that inequalities could be reduced by strengthening information and service provision by community and village health workers to promote and sustain timely care-seeking. Finally, larger inequalities for interventions that require a fully functioning health system suggest that investments in health facilities and human resources, particularly in areas that are disproportionately inhabited by the poor and ethnic minorities, may contribute to reducing inequalities.

## Background

Vietnam has achieved considerable success in economic development, poverty reduction, and health over a relatively short period of time. From being one of the poorest countries in the world in the early 1980s, it has become a lower-middle-income country with an estimated gross national income (GNI) per capita of US$ 1,160 in 2010 [1]. The poverty headcount decreased from 37% in 1998 to 14% in 2008. The under-five mortality rate decreased from 51 deaths per 1,000 live births in 1990 to 23 in 2010 and the maternal mortality ratio decreased from 170 to 59 deaths per 100,000 live births in the same time period, putting Vietnam on track to achieve Millennium Development Goals (MDGs) 4 and 5 on child and maternal health [2,3].

However, these improvements have not been spread equally across the population. Several studies have found significant, sustained and sometimes widening disparities in health outcomes between population groups [4,5]. In response to these disparities, the government launched a program in 2003 called the Health Care Fund for the Poor to increase access to health services and reduce the financial burden of health care by providing free health insurance to the poor, ethnic minorities and children under six years old. By 2007, the number of beneficiaries was about 15 million, equal to around 18% of the total population [6]. Two recent studies of the impact of the initial phase of this program found mixed effects on health care utilization and out-of-pocket spending by beneficiaries of the program, but findings were promising enough to justify further financial and political support in combination with efforts to strengthen operational aspects of the program [7,8].

Vietnam is not unique in displaying inequalities in reproductive, maternal, newborn and child health (RMNCH). Several single- and multi-country studies have demonstrated that poor mothers and children do worse than their better-off peers in health outcomes, access to health care, and payment for health care as a share of income [9-12]. Several studies have explored how differences in outcomes are associated with factors such as knowledge of preventive measures, coverage of health services, and access to financial protection mechanisms [13-15]. Most of these studies have measured inequalities by living standards, as measured by income, expenditure, consumption or wealth. Fewer studies have studies inequalities by other dimensions, such as education [16], ethnicity [17], place of residence [18], and gender [19].

The objectives of this study are two-fold: (i) to explore whether or not inequalities in RMNCH and nutrition outcomes and intervention coverage changed over time in Vietnam in 1997–2006, and (ii) to analyze if inequalities were different depending on the type of stratifying variable used to measure inequalities and on the type of outcome studied. Two main contributions to the literature are suggested: (i) to our knowledge, this is the first analysis of long-term trends in RMNCH inequalities in Vietnam using nationally representative data, and (ii) analyzing inequalities by several socioeconomic dimensions in addition to living standards will contribute to an increased understanding of the range of factors that influence inequalities in RMNCH.

## Methods

### Data sources

We used publicly available data collected at four points in time: 1997 and 2002 cross-sectional data from the Demographic and Health Surveys (DHS) and 2000 and 2006 cross-sectional data from the Multiple Indicator Cluster Survey (MICS) [20,21]. The DHS and the MICS collect comparable, nationally representative data on health and nutrition outcomes, intervention coverage, demographic information and a wealth index. Using data from both DHS and MICS to analyze trends in health outcomes and service coverage is common in the literature [22-25]. Each survey contains information provided by women of reproductive age (15–49 years old) from at least 7,000 households (1997: 7,001; 2000: 7,628; 2002: 7,048; 2006: 8,355; each with a response rate of at least 98%). As a consequence of the stratified sampling survey design used by both DHS and MICS, different observations have different probabilities of selection. We adjusted for this by applying weights to each observation equal to the inverse of the probability of being sampled [26].

### Health and nutrition outcome indicators

DHS and MICS contain information required for estimating infant and under-five mortality rates, malnutrition and prevalence of common child illnesses (see Table 1 for a list of the health and nutrition outcome indicators included in the study).

**Table 1:** Indicators of health and nutrition outcomes used in the study

**Category and sub-category**	**Indicator**
**1. Health outcomes**	
Infant mortality	Number of deaths of infants aged 0–12 months per 1,000 live births
Under-five mortality	Number of deaths of children aged 0–59 months per 1,000 live births
Childhood illness	% of children aged 0–59 months with suspected acute respiratory infection in last 2 weeks
	% of children aged 0–59 months with diarrhea in last 2 weeks
	% of children aged 0–59 months with fever in last 2 weeks
**2. Nutrition outcomes**	
Low birth weight	% of births with weight less than 2,500 g
Child malnutrition	% of children aged 0–5 years who are underweight (weight for age is more than two standard deviations [SD] below the median of an international reference population)
	% of children aged 0–5 years who are stunted (height for age is more than two SD below the median of an international reference population)
	% of children aged 0–5 years who are wasted (weight for height is more than two SD below the median of an international reference population)

The two data sources we use in this study call for two different methods of estimating infant and under-five mortality. DHS surveys collect full birth histories from mothers, which enables direct estimation of mortality [27]. Using this approach we constructed life-tables with survival times, which were then used to calculate mortality rates. MICS surveys collect incomplete birth histories, which include data on the number of children born and the number of children surviving, but no information on dates of these events. Incomplete birth histories require indirect estimation of mortality, which involves applying a model life table to the available data, i.e. number of children ever born and number of children still alive [28]. We used the QFIVE software to calculate mortality rates with the indirect estimation method [29].

We calculated prevalence of pneumonia and diarrhea, which remain the leading killers of children globally; in 2008 they together accounted for one-third of deaths among children less than five years of age [30]. To measure inequalities in malnutrition, which contributes to one-third of child deaths globally, we calculated prevalence of underweight, stunting and wasting [31]. Only the 2000 MICS survey included data on these three indicators, which prevented a trend analysis. However, all four surveys included data on low birth-weight, which enabled us to study trends in an indicator considered an important predictor of a newborn’s survival, growth, and long-term health and psychosocial development, as well as being associated with a pregnant woman's overall health status [32].

### Intervention coverage indicators

Although the pathways to child mortality and morbidity are complex and influenced by economic development and other social determinants of health, interventions in the health system contribute to improving health outcomes [33]. A recent systematic review of the evidence has identified 56 interventions that are key to improving RMNCH outcomes [34]. It has been estimated that if these interventions were universally available, they could reduce global child mortality by two-thirds and maternal deaths by at least half [35,36]. It is therefore of interest to study inequalities in coverage of these interventions (see Table 2 for a list of the intervention coverage indicators for which data were available in our data sources, and their definitions, included in the study).

**Table 2:** Indicators of intervention coverage used in the study

**Category and sub-category**	**Indicator**
**1. Family planning**	
Any method	% of women using any family planning method
Modern method	% of women using a modern family planning method (contraceptive pill, intrauterine device, injections, condom, sterilization)
**2. Maternal and newborn health**	
Antenatal care	% of pregnant women attended at least once for antenatal care during pregnancy by skilled health personnel
	% of pregnant women attended at least 4 times for antenatal care during pregnancy by skilled health personnel
Tetanus toxoid vaccination	% of women who received at least 2 tetanus toxoid doses during last pregnancy (if in last year)
Place of delivery	% of births delivered in health facilities
Skilled birth attendance	% of deliveries attended by skilled health personnel (doctor/nurse/midwife)
**3. Child health**	
Feeding	% of children born in the last 12 months who were breastfed within one hour of birth
	% of infants under 6 months exclusively breastfed
	% of infants aged 6–9 months who are breastfed and receive complementary food
Immunization	% of children aged 12–23 months who are immunized against measles
	% of children aged 12–23 months who received BCG vaccine
	% of children aged 12–23 months who received three doses of DPT vaccine
Vitamin A supplementation	% of children 6 to 59 months receiving one dose of Vitamin A in the past 6 months
Careseeking and treatment of common childhood illnesses	% of children with diarrhea in the last 2 weeks who were brought to an appropriate health provider
	% of children aged 0–59 months with diarrhea in the last 2 weeks who received ORT and continued feeding
	% of children with suspected acute respiratory infection in the last 2 weeks who were brought to an appropriate health provider
	% of children aged 0–59 months with suspected ARI receiving antibiotics

### Inequality stratifying variables

We measured inequalities by several stratifying variables. Direct measures of living standards such as income, expenditure and consumption are rarely collected in health surveys such as DHS and MICS. However, both DHS and MICS collect data on living conditions and household assets, which can be used to compute an indirect or proxy measure for living standards called the asset or wealth index [37,38]. The index is calculated for each household in a survey to divide the sample into quintiles: five groups of equal size, from poorest to richest.

We also estimated inequalities by education of mother, ethnicity, region, urban versus rural residence, and sex of the child. The two surveys use a different number of categories for the highest level of education obtained by the mother. MICS uses seven categories in the 2000 survey and five in 2006. DHS uses six categories in both 1997 and 2002. To address this we constructed four categories for education of mother: no education, primary education, lower secondary education, and upper secondary and higher education.

Since there are more than 50 ethnic minority groups in Vietnam, comprising 14% of the total population, we combined them into one group and compare their outcomes with those of the majority ethnic group [39]. We also assessed inequalities by urban versus rural residence and by region. MICS uses eight regions, while DHS uses seven regions. To adjust for this we obtained the list of provinces included in the MICS regions and then allocated those provinces in the same manner as was done in the DHS, leaving us with seven regions for analysis in both surveys.

### Measures of inequality

We used several complementary methods to study inequalities in MNCH, all of which have found broad acceptance in the health inequality literature [40]. We tabulated health and nutrition outcomes and intervention coverage by stratifying variable, for example under-five mortality by wealth quintile and immunization coverage for the seven regions. We calculated ratios for health and nutrition outcomes and intervention coverage, such as poorest vs. richest quintile, ethnic minority vs. majority, and rural vs. urban place of residence. These measures of inequality provide a broad descriptive analysis of inequalities in MNCH. However, while tabulations and ratios are intuitive in their presentation, they have certain limitations. For example, ratios ignore patterns in the middle of the wealth and education distributions and tabulations do not shed light on variations within quintiles or education levels. A more comprehensive picture of inequality across the full distribution is provided by the concentration index (CI), which is a measure of the magnitude of inequality that can be compared across time, country and region [41]. has been used to compare inequality in child mortality [42], child immunization and child malnutrition [43]. The CI is defined as twice the area between the concentration curve and the line of equality.^a^ The CI varies from −1 to 1, with 0 indicating perfect equality. The index takes a negative value if it lies above the line of equality, which indicates a disproportionate concentration of the variable among the poor. If the CI of mortality is negative it means that mortality is higher among the poor. It takes a positive value if it lies below the line of equality, indicating that the variable is disproportionately concentrated among the rich. If the CI for immunization is positive, it means that the rich benefit from higher coverage of this intervention. The CI requires data that can be ranked in a meaningful way. This requirement holds for measures of living standards, such as the wealth index, and education levels, but there is no way to meaningfully rank individuals or households by ethnicity, geography, or gender. We were therefore limited in generating concentration indices for health and nutrition outcomes and intervention coverage indicators for the living standard and education stratifying variables. To examine trends in inequality over time, we tested the null hypothesis that there was no change between 1997 and 2006.

With the exception of mortality estimates from MICS data, which were computed using the QFIVE software, Stata version 10.0 was used for all analyses of this study.

## Results

### Health and nutrition outcomes

The mean infant mortality rate (IMR) decreased from 32.0 deaths per 1,000 live births in 1997 to 18.8 in 2006, while the mean under-five mortality rate (U5MR) decreased from 41.2 to 23.0. There were inequalities in mortality rates by living standards (see Table 3). For example, in 1997 a child in the poorest 20% of the population was almost three times (2.8) more likely to die before his or her fifth birthday compared to his or her peers in the richest 20% of the population. These inequalities increased over time; in 2006 the poor/rich ratio was 4.5. Similar inequalities were identified for the IMR.

**Table 3:** Infant and under-five mortality rates in Vietnam, 1997–2006

	**IMR**				**U5MR**			
**Year**	**1997**	**2000**	**2002**	**2006**	**1997**	**2000**	**2002**	**2006**
**1. Total sample**	32.0	36.8	24.2	18.8	41.2	47.0	30.3	23.0
**2. Living standards**								
Poorest quintile	42.4	45.5	33.6	19.8	61.0	59.5	43.9	24.0
2nd quintile	37.5	38.0	29.2	23.5	45.8	48.0	34.4	29.3
Middle quintile	30.7	35.5	20.8	22.5	33.4	45.0	25.6	26.8
4^th^ quintile	28.7	26.3	19.0	14.3	37.0	32.3	25.8	17.8
Richest quintile	15.1	14.0	12.7	4.4	20.7	17.3	14.2	5.6
Ratio poor/rich	2.8	3.3	2.6	4.5	2.9	3.4	3.1	4.3
Concentration Index	−0.1639	−0.1913	−0.1764	−0.1896	−0.1828	−0.2024	−0.1843	−0.1875
p (CI = 0)	p < 0.05	p < 0.05	p < 0.05	p > 0.05	p < 0.05	p < 0.05	p < 0.05	p > 0.05
p (CI_1997_ = CI_2006_)	p > 0.05	p > 0.05	p > 0.05	p > 0.05	p > 0.05	p > 0.05	p > 0.05	p > 0.05
**3. Education of mother**								
No education	38.8	47.5	25.9	32.3	57.0	63.0	35.6	41.0
Primary	38.7	40.0	29.5	17.3	50.9	51.5	34.3	20.8
Lower secondary	31.4	33.5	21.9	24.0	39.8	42.0	29.1	30.0
Upper secondary or higher	17.4	12.3	18.8	5.6	20.7	14.8	21.7	6.7
Ratio no education / upper secondary or higher	2.2	3.9	1.4	5.8	2.8	4.3	1.6	6.1
Concentration Index	−0.1071	−0.1685	−0.0785	−0.1137	−0.1278	−0.1809	−0.0734	−0.1157
p (CI = 0)	p > 0.05	p > 0.05	p < 0.05	p > 0.05	p > 0.05	p > 0.05	p < 0.05	p > 0.05
p (CI_1997_ = CI_2006_)	p > 0.05	p > 0.05	p > 0.05	p > 0.05	p > 0.05	p > 0.05	p > 0.05	p > 0.05
**4. Ethnicity**								
Majority	27.0	32.0	23.2	15.8	34.7	40.0	28.4	18.8
Minority	55.6	44.5	28.5	33.8	72.5	58.3	38.5	43.0
Ratio minority / majority	2.1	1.4	1.2	2.1	2.1	1.5	1.4	2.3
**5. Sex of child**								
Male	39.7	36.3	25.6	14.0	49.1	46.3	33.4	17.5
Female	23.5	36.0	22.7	20.8	32.6	46.3	27.0	25.5
Ratio female/ male	0.6	1.0	0.9	1.5	0.7	1.0	0.8	1.5
**6. Place of residence**								
Urban	20.6	18.5	13.3	3.8	25.1	22.5	15.9	4.5
Rural	34.3	38.3	26.5	22.0	44.6	48.5	33.3	27.3
Ratio rural/ urban	1.7	2.1	2.0	5.9	1.8	2.2	2.1	6.1
**7. Region**								
Red River Delta	25.1	39.3	22.4	29.8	32.3	50.0	27.2	37.3
Northern Uplands	40.7	35.0	28.2	29.0	50.2	44.0	32.9	37.0
North Central Coast	35.0	39.3	40.1	18.8	40.0	50.8	47.1	22.5
South Central Coast	31.4	32.5	17.8	10.8	40.8	41.0	19.8	13.0
Central Highlands	45.0	37.0	21.8	17.8	51.6	47.5	35.8	21.5
Southeast	17.8	13.0	11.0	6.8	17.8	16.0	19.5	8.5
Mekong River Delta	33.1	45.0	21.5	12.3	51.5	59.3	28.8	15.0

Notes:

1. Infant mortality rate (IMR) = number of deaths of children less than one year of age per 1,000 live births.

2. Under-five mortality rate (U5MR) = number of deaths of children less than five years of age per 1,000 live births.

3. p (CI = 0) = probability of concentration index being equal to zero (if p < 0.05, then there is a statistically significant difference between a concentration index of zero, indicating perfect equality, and the obtained concentration index; such a result indicates inequality).

4. p (CI_1997_ = CI_2006_) = probability of concentration index for 1997 being equal to concentration index for 2006 (if p < 0.05, then there is a statistically significant difference between the concentration index in 1997 and the concentration index in 2006; such a result indicates a change in inequality over time).

**Figure 1:**
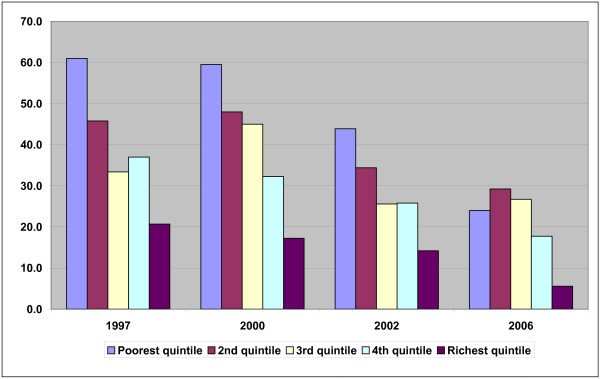
Under-five mortality rates in Vietnam 1997–2006 (number of deaths of children less than five years of age per 1,000 live births).

The largest reductions for both IMR and U5MR in 1997–2006 were recorded in the richest and poorest quintiles; the middle quintiles recorded smaller reductions. Within each survey the mortality rate generally decreased from the poorest quintile to the 2^nd^ quintile and so on, with a few exceptions (see Figure 1).

The CI confirmed the living standards inequalities identified through tabulations and ratios. The CI in 1997 was −0.1639 for IMR and −0.1828 for U5MR. By 2006, disparities had increased to −0.1896 for IMR and to −0.1875 for U5MR.

While smaller in magnitude than for living standards, there were inequalities by the other stratifying variables as well (see Table 3). In 2006, the U5MR for children whose mother had no education was 41.0 deaths per 1,000 live births, while the rate for children whose mother had completed upper secondary education or higher was 6.7. In 1997, the U5MR of ethnic majority and minorities groups were 34.7 and 72.5 deaths per 1,000 live births, respectively (a ratio of 2.1). This inequality increased slightly over time; in 2006 the ratio was 2.3.

The analysis of differences by sex of the child found that boys were more likely to die before their fifth birthday in 1997, 2000 and 2002.The U5MR for boys was 49.1 deaths per 1,000 live births in 1997; for girls it was 32.6. In 2006, inequalities ran in the opposite direction; the rate was 17.5 for boys and 25.5 for girls. There were also geographic inequalities. The IMR and U5MR were about twice as high for children living in rural versus urban areas in 1997–2002; in 2006 it was about six times as high. In 2006, mortality rates were highest in the Northern Uplands and Red River Delta. The largest reductions in under-five mortality between 1997 and 2006 occurred in the Mekong River Delta.

Inequalities in the three nutrition outcomes for which we only had data from the 2000 MICS survey were identified for all stratifying variables (see Table 4). The poor/rich ratio was 2.75 for stunting, 2.41 for underweight and 1.49 for wasting. The CI in 2000 was −0.1067 for underweight, -0.1216 for stunting, and −0.0967 for wasting. Almost half (46.5%) of children with mothers without education were underweight, while the figure for upper secondary or higher education was 20.0%. Nutrition inequalities were less pronounced by ethnicity, urban versus rural residence, and sex of child.

**Table 4:** Nutrition outcomes in Vietnam, 1997–2006

	**Percentage of children under five who are under-weight**	**Percentage of children under five who are stunted**	**Percentage of children under five who are wasted**	**Percentage of newborn with low birth weight (<2,500 g)**	**Percentage of newborn with low birth weight (<2,500 g)**	**Percentage of newborn with low birth weight (<2,500 g)**	**Percentage of newborn with low birth weight (<2,500 g)**
**Year**	**2000**	**2000**	**2000**	**1997**	**2000**	**2002**	**2006**
**1. Total sample**	32.4	35.9	5.6	8.9	5.7	7.0	6.2
**2. Living standards**							
Poorest quintile	41.0	44.4	7.4	17.2	8.0	11.3	9.6
2^nd^ quintile	35.7	41.8	4.8	12.4	6.3	6.9	6.6
Middle quintile	30.6	37.0	4.2	7.7	7.8	6.3	6.1
4^th^ quintile	28.7	30.8	5.0	5.7	5.8	8.4	6.8
Richest quintile	17.0	16.1	5.0	4.0	1.6	3.5	3.7
Ratio poor/rich	2.41	2.75	1.49	4.26	5.01	3.21	2.58
Concentration Index	−0.1067	−0.1216	−0.0967	−0.2810	−0.2081	−0.1584	−0.1236
p (CI = 0)	p < 0.05	p < 0.05	p > 0.05	p < 0.05	p > 0.05	p < 0.05	p > 0.05
p (CI_1997_ = CI_2006_)	N/a	N/a	N/a	p > 0.05	p > 0.05	p > 0.05	p > 0.05
**3. Education of mother**							
No education	46.5	55.5	5.2	14.2	6.9	17.7	6.4
Primary	36.8	40.1	6.4	10.0	6.5	6.9	5.6
Lower secondary	30.5	34.2	5.3	8.5	4.3	6.8	7.4
Upper secondary or higher	20.0	19.5	5.3	5.2	7.2	3.1	5.6
Ratio no education / upper secondary or higher	2.32	2.84	0.97	2.74	0.96	5.70	1.15
Concentration Index	−0.0816	−0.1254	−0.0336	−0.1063	0.1238	−0.1684	−0.0040
p (CI = 0)	p < 0.05	p < 0.05	p > 0.05	p > 0.05	p > 0.05	p < 0.05	p > 0.05
p (CI_1997_ = CI_2006_)	N/a	N/a	N/a	p > 0.05	p > 0.05	p > 0.05	p > 0.05
**4. Ethnicity**							
Majority	29.4	32.0	5.3	7.9	6.0	6.2	6.1
Minority	44.9	52.2	6.9	18.7	2.0	14.4	6.8
Ratio minority / majority	1.53	1.63	1.31	2.36	0.33	2.35	1.11
**5. Sex of child**							
Male	30.5	34.8	6.3	7.2	N/a	6.8	N/a
Female	34.3	37.1	4.8	10.7	N/a	7.2	N/a
Ratio female / male	1.12	1.07	0.75	1.49	N/a	1.06	N/a
**6. Place of residence**							
Urban	21.1	19.2	6.0	4.3	1.7	4.0	3.5
Rural	35.0	39.8	5.5	10.2	7.1	7.8	7.2
Ratio rural/urban	1.66	2.08	0.91	2.36	4.18	1.96	2.02
**7. Region**							
Red River Delta	27.5	34.4	4.7	6.5	3.8	5.0	7.8
Northern Uplands	37.1	48.0	3.6	5.3	1.0	8.0	6.3
North Central Coast	38.4	44.8	6.4	11.2	12.2	3.6	5.0
South Central Coast	30.2	31.5	5.1	9.0	7.0	7.1	5.0
Central Highlands	43.8	43.2	6.6	3.2	3.7	21.0	6.1
Southeast	26.8	25.8	5.0	8.7	7.2	7.6	4.7
Mekong River Delta	28.6	25.4	8.2	13.1	6.6	7.1	6.8

Notes:

1. N/a = not applicable or data not available.

2. p (CI = 0) = probability of concentration index being equal to zero (if p < 0.05, then there is a statistically significant difference between a concentration index of zero, indicating perfect equality, and the obtained concentration index; such a result indicates inequality).

3. p (CI_1997_ = CI_2006_) = probability of concentration index for 1997 being equal to concentration index for 2006 (if p < 0.05, then there is a statistically significant difference between the concentration index in 1997 and the concentration index in 2006; such a result indicates a change in inequality over time).

Inequalities by living standards for low birth weight were considerable, although they decreased over time. For example, the CI for low birth weight declined from −0.2810 to −0.1236 between 1997 and 2006. Inequalities by education (CI = −0.0040) and ethnicity (minority/majority ratio = 1.11) had almost disappeared by 2006.

Mixed results were found for child illness inequalities by living standards as measured by the CI (see Table 5). The CI for proportion of children with suspected acute respiratory infection decreased from −0.0794 in 1997 to −0.0231 in 2006, while the CI for proportion of children with diarrhea increased from −0.0603 in 1997 to −0.1414 in 2006. We also found inequalities by the other stratifying variables, but they were generally less pronounced. We did not identify any strong patterns of change over time in inequalities in prevalence of child illness by education, ethnicity, region, urban versus rural residence, or sex of child.

**Table 5:** Prevalence of child illness in Vietnam in the two weeks preceding the survey, 1997–2006

	**Percentage of children less than five years of age with suspected acute respiratory infection**				**Percentage of children less than five years of age with diarrhea**			
**Year**	**1997**	**2000**	**2002**	**2006**	**1997**	**2000**	**2002**	**2006**
**1. Total sample**	14.2	9.3	19.6	6.5	10.1	11.3	11.4	6.8
**2. Living standards**								
Poorest quintile	14.0	11.9	23.8	4.9	10.2	17.5	18.3	9.5
2^nd^ quintile	18.2	11.5	21.6	6.3	11.2	11.0	12.5	6.7
Middle quintile	15.9	7.7	19.8	9.2	12.0	9.3	12.1	6.8
4^th^ quintile	9.8	7.9	16.9	9.0	9.5	7.9	7.3	6.9
Richest quintile	10.2	4.1	14.0	3.1	6.2	5.5	3.8	4.4
Poor/rich ratio	1.38	2.88	1.70	1.59	1.65	3.19	4.81	2.19
Concentration Index	−0.0794	−0.1680	−0.1111	−0.0231	−0.0604	−0.2235	−0.2577	−0.1414
p (CI = 0)	p < 0.05	p < 0.05	p < 0.05	p > 0.05	p > 0.05	p < 0.05	p < 0.05	p < 0.05
p (CI_1997_ = CI_2006_)	p > 0.05	p > 0.05	p > 0.05	p > 0.05	p > 0.05	p > 0.05	p > 0.05	p > 0.05
**3. Education of mother**								
No education	15.6	8.4	15.5	6.1	12.5	21.0	16.1	8.2
Primary	12.9	12.0	21.3	6.0	11.9	13.1	11.9	7.8
Lower secondary	15.2	9.8	22.0	8.3	8.9	10.1	11.8	5.7
Upper secondary or higher	13.1	4.5	12.1	5.2	8.1	4.8	6.1	5.3
Ratio no education / upper secondary or higher	1.19	1.84	1.28	1.19	1.54	4.41	2.63	1.54
Concentration Index	−0.0045	−0.0958	−0.0638	0.0418	−0.0764	−0.1904	−0.1061	−0.0722
p (CI = 0)	p > 0.05	p < 0.05	p > 0.05	p > 0.05	p > 0.05	p < 0.05	p < 0.05	p > 0.05
p (CI_1997_ = CI_2006_)	p > 0.05	p > 0.05	p > 0.05	p > 0.05	p > 0.05	p > 0.05	p > 0.05	p > 0.05
**4. Ethnicity**								
Majority	13.3	9.3	17.5	6.8	9.8	9.5	9.0	5.9
Minority	17.3	9.2	28.4	5.2	11.1	19.0	21.2	11.0
Ratio minority/ majority	1.30	0.99	1.62	0.77	1.13	1.99	2.36	1.85
**5. Sex of child**								
Male	16.9	9.1	21.9	6.5	11.7	12.5	12.7	7.5
Female	11.3	9.4	17.0	6.4	8.4	10.1	9.9	6.1
Ratio female/ male	0.67	1.04	0.78	0.98	0.71	0.81	0.78	0.81
**6. Place of residence**								
Urban	11.1	5.2	14.0	4.3	5.8	6.3	3.5	4.2
Rural	14.7	10.2	20.8	7.2	10.8	12.5	13.0	7.7
Ratio rural/ urban	1.33	1.96	1.48	1.68	1.86	2.00	3.75	1.84
**7. Region**								
Red River Delta	19.7	6.5	18.2	10.0	10.3	6.8	7.5	8.9
Northern Uplands	18.0	10.8	28.8	4.8	11.2	15.9	17.7	6.8
North Central Coast	10.8	14.0	16.7	8.6	10.6	12.2	8.9	5.4
South Central Coast	8.3	10.7	21.8	6.3	11.2	11.8	18.6	6.4
Central Highlands	11.1	7.6	21.0	7.3	8.1	14.7	15.3	10.1
Southeast	10.3	4.9	13.4	4.6	4.1	6.7	5.2	6.3
Mekong River Delta	14.7	9.4	16.1	4.6	11.8	12.2	8.5	5.7

Notes:

1. p (CI = 0) = probability of concentration index being equal to zero (if p < 0.05, then there is a statistically significant difference between a concentration index of zero, indicating perfect equality, and the obtained concentration index; such a result indicates inequality).

2. p (CI_1997_ = CI_2006_) = probability of concentration index for 1997 being equal to concentration index for 2006 (if p < 0.05, then there is a statistically significant difference between the concentration index in 1997 and the concentration index in 2006; such a result indicates a change in inequality over time).

### Intervention coverage

The results for intervention coverage are presented in Table 6. We found small inequalities for family planning interventions in 1997–2006. Almost all maternal and newborn health indicators displayed decreasing inequalities by both living standards and education over time. For example, for at least one antenatal care contact the living standard CI decreased from 0.2833 in 1997 to 0.0587 in 2006, while the education CI decreased from 0.0765 to 0.0463. Inequalities were more pronounced for interventions requiring multiple service contacts. For example, while pregnant women in the richest quintile were 1.5 times more likely than pregnant women in the poorest quintile to have been attended at least once during pregnancy by skilled health personnel in 2002 (CI = 0.0776), they were almost five times as likely to have been attended at least four times (CI = 0.3170). In 2006, pregnant women in the poorest quintile were about half as likely as the rich to have delivered in a health facility (CI = 0.1182) and to have had their births attended by skilled health personnel (CI = 0.1025). These indicators exhibited considerable inequalities by other stratifying variables as well: the CI by education for delivery in facility and skilled birth attendance were 0.0669 and 0.0612, respectively, in 2006. The ethnic minority/majority ratios for these two interventions were 0.43 and 0.47, respectively.

**Table 6:** Concentration index for coverage of MNCH interventions in Vietnam, 1997–2006

**Intervention**	**Wealth score**				**Maternal education**			
	**1997**	**2000**	**2002**	**2006**	**1997**	**2000**	**2002**	**2006**
**1. Family planning**								
% of women using any type of family planning method	0.0384	0.0432	0.0249	−0.0054	0.0335	0.0487	0.0315	0.0123
p (CI = 0)	p < 0.05	p < 0.05	p < 0.05	p > 0.05	p < 0.05	p < 0.05	p < 0.05	p < 0.05
p (CI_1997_ = CI_2006_)	p < 0.05	p < 0.05	p < 0.05	p < 0.05	p < 0.05	p < 0.05	p < 0.05	p < 0.05
% of women using modern type of family planning method	0.022	0.0074	−0.0066	−0.0348	0.0209	0.0437	0.0075	−0.0004
p (CI = 0)	p < 0.05	p > 0.05	p > 0.05	p < 0.05	p < 0.05	p < 0.05	p > 0.05	p > 0.05
p (CI_1997_ = CI_2006_)	p < 0.05	p < 0.05	p < 0.05	p < 0.05	p < 0.05	p < 0.05	p < 0.05	p < 0.05
**2. Maternal and newborn health**								
% of pregnant women attended at least once during pregnancy by skilled health personnel	0.2833	0.1652	0.0776	0.0587	0.0765	0.1428	0.0523	0.0463
p (CI = 0)	p < 0.05	p < 0.05	p < 0.05	p < 0.05	p < 0.05	p < 0.05	p < 0.05	p < 0.05
p (CI_1997_ = CI_2006_)	p < 0.05	p < 0.05	p < 0.05	p < 0.05	p < 0.05	p < 0.05	p < 0.05	p < 0.05
% of pregnant women attended at least 4 times during pregnancy by skilled health personnel	0.4296	N/a	0.3170	N/a	0.2101	N/a	0.1741	N/a
p (CI = 0)	p < 0.05	N/a	p < 0.05	N/a	p < 0.05	N/a	p < 0.05	N/a
p (CI_1997_ = CI_2006_)	N/a	N/a	N/a	N/a	N/a	N/a	N/a	N/a
% of women who received at least two tetanus toxoid doses during last pregnancy	0.1373	0.1119	0.0952	0.0745	0.0690	0.1238	0.0536	0.0487
p (CI = 0)	p < 0.05	p < 0.05	p < 0.05	p < 0.05	p < 0.05	p < 0.05	p < 0.05	p < 0.05
p (CI_1997_ = CI_2006_)	p < 0.05	p < 0.05	p < 0.05	p < 0.05	p < 0.05	p < 0.05	p < 0.05	p < 0.05
% of births delivered in health facilities	0.1964	N/a	0.1324	0.1182	0.0561	N/a	0.0721	0.0669
p (CI = 0)	p < 0.05	N/a	p < 0.05	p < 0.05	p < 0.05	N/a	p < 0.05	p < 0.05
p (CI_1997_ = CI_2006_)	p < 0.05	p < 0.05	p < 0.05	p < 0.05	p > 0.05	p > 0.05	p > 0.05	p > 0.05
% of deliveries attended by skilled health personnel	0.1407	0.1760	0.1247	0.1025	0.0570	0.1490	0.0771	0.0612
p (CI = 0)	p < 0.05	p < 0.05	p < 0.05	p < 0.05	p < 0.05	p < 0.05	p < 0.05	p < 0.05
p (CI_1997_ = CI_2006_)	p < 0.05	p < 0.05	p < 0.05	p < 0.05	p > 0.05	p > 0.05	p > 0.05	p > 0.05
% of children born in the last 12 months who were breastfed within one hour of birth	0.0218	N/a	−0.0075	−0.0244	0.0383	N/a	0.0286	0.0227
p (CI = 0)	p > 0.05	N/a	p > 0.05	p > 0.05	p < 0.05	N/a	p < 0.05	p > 0.05
p (CI_1997_ = CI_2006_)	p > 0.05	p > 0.05	p > 0.05	p > 0.05	p > 0.05	p > 0.05	p > 0.05	p > 0.05
% of infants under 6 months exclusively breastfed	−0.0599	N/a	−0.1062	−0.1472	0.0345	N/a	0.0460	−0.1151
p (CI = 0)	p > 0.05	N/a	p < 0.05	p < 0.05	p < 0.05	N/a	p > 0.05	p < 0.05
p (CI_1997_ = CI_2006_)	p > 0.05	p > 0.05	p > 0.05	p > 0.05	p > 0.05	p > 0.05	p > 0.05	p > 0.05
% of infants aged 6–9 months who are breastfed and receive complementary food	−0.0160	−0.0352	−0.0377	−0.0063	0.0194	−0.0271	−0.0663	−0.0503
p (CI = 0)	p > 0.05	p > 0.05	p > 0.05	p > 0.05	p > 0.05	p > 0.05	p < 0.05	p > 0.05
p (CI_1997_ = CI_2006_)	p > 0.05	p > 0.05	p > 0.05	p > 0.05	p > 0.05	p > 0.05	p > 0.05	p > 0.05
**3. Child health**								
% of children aged 0–59 months who are immunized against measles	0.0698	0.0775	0.0718	0.0341	0.0575	0.0778	0.0394	0.0211
p (CI = 0)	p < 0.05	p < 0.05	p < 0.05	p < 0.05	p < 0.05	p < 0.05	p < 0.05	p < 0.05
p (CI_1997_ = CI_2006_)	p < 0.05	p < 0.05	p < 0.05	p < 0.05	p < 0.05	p < 0.05	p < 0.05	p < 0.05
% of children aged 0–59 months who received BCG vaccine	0.0513	0.0685	0.0432	0.0164	0.0184	0.0580	0.0244	0.0121
p (CI = 0)	p < 0.05	p < 0.05	p < 0.05	p < 0.05	p < 0.05	p < 0.05	p < 0.05	p < 0.05
p (CI_1997_ = CI_2006_)	p < 0.05	p < 0.05	p < 0.05	p < 0.05	p > 0.05	p > 0.05	p > 0.05	p > 0.05
% of children aged 0–59 months who received 3 doses of DPT vaccine	0.0844	0.1334	0.1051	0.0641	0.0587	0.1164	0.0602	0.0539
p (CI = 0)	p < 0.05	p < 0.05	p < 0.05	p < 0.05	p < 0.05	p < 0.05	p < 0.05	p < 0.05
p (CI_1997_ = CI_2006_)	p > 0.05	p > 0.05	p > 0.05	p > 0.05	p > 0.05	p > 0.05	p > 0.05	p > 0.05
% of children aged 0–59 months receiving one dose of Vitamin A in the past 6 months	N/a	0.0765	N/a	0.0436	N/a	0.0559	N/a	0.0393
p (CI = 0)	N/a	p < 0.05	N/a	p < 0.05	N/a	p < 0.05	N/a	p < 0.05
p (CI_1997_ = CI_2006_)	N/a	N/a	N/a	N/a	N/a	N/a	N/a	N/a
% of children aged 0–59 months with diarrhea in the last 2 weeks who were brought to an appropriate health provider	0.0119	0.0796	0.0884	0.0101	−0.0612	0.0749	0.0736	0.0685
p (CI = 0)	p > 0.05	p < 0.05	p < 0.05	p > 0.05	p > 0.05	p < 0.05	p > 0.05	p < 0.05
p (CI_1997_ = CI_2006_)	p > 0.05	p > 0.05	p > 0.05	p > 0.05	p > 0.05	p > 0.05	p > 0.05	p > 0.05
% of children aged 0–59 months with diarrhea in the last 2 weeks who received ORT and continued feeding	0.1887	0.0059	0.1088	−0.0526	−0.0042	0.0425	−0.0215	−0.0193
p (CI = 0)	p < 0.05	p > 0.05	p > 0.05	p > 0.05	p > 0.05	p > 0.05	p > 0.05	p > 0.05
p (CI_1997_ = CI_2006_)	p < 0.05	p < 0.05	p < 0.05	p < 0.05	p > 0.05	p > 0.05	p > 0.05	p > 0.05
% of children aged 0–59 months with suspected ARI in the last 2 weeks who were brought to an appropriate health provider	0.0619	0.0755	0.0161	0.0579	0.0393	0.0931	0.0532	0.0780
p (CI = 0)	p < 0.05	p < 0.05	p > 0.05	p < 0.05	p > 0.05	p < 0.05	p < 0.05	p < 0.05
p (CI_1997_ = CI_2006_)	p > 0.05	p > 0.05	p > 0.05	p > 0.05	p > 0.05	p > 0.05	p > 0.05	p > 0.05
% of children aged 0–59 months with suspected ARI receiving antibiotics	N/a	N/a	N/a	0.0135	N/a	N/a	N/a	0.0088
p (CI = 0)	N/a	N/a	N/a	p > 0.05	N/a	N/a	N/a	p > 0.05
p (CI_1997_ = CI_2006_)	N/a	N/a	N/a	N/a	N/a	N/a	N/a	N/a

Notes:

1. N/a = not applicable or data not available.

2. p (CI = 0) = probability of concentration index being equal to zero (if p < 0.05, then there is a statistically significant difference between a concentration index of zero, indicating perfect equality, and the obtained concentration index; such a result indicates inequality).

3. p (CI_1997_ = CI_2006_) = probability of concentration index for 1997 being equal to concentration index for 2006 (if p < 0.05, then there is a statistically significant difference between the concentration index in 1997 and the concentration index in 2006; such a result indicates a change in inequality over time).

Two measures exhibited pro-poor inequalities: early initiation of breastfeeding and exclusive breastfeeding. For example, in 2006, women in the poorest quintile of the population were more than twice as likely as women in the richest quintile to exclusively breastfeed their children until they reached six months of age (CI = −0.1472). A similar pattern was found for education (CI = −0.1151) and ethnicity (minority/majority ratio = 1.68).

We found inequalities for interventions targeted to children less than five years of age, but they were less pronounced than for maternal and newborn health interventions. Almost all child health indicators displayed decreasing inequalities by both living standards and education over time. For example, for measles vaccination the living standard CI decreased from 0.0698 in 1997 to 0.0341 in 2006, while the education CI decreased from 0.0575 to 0.0211. Interventions that require multiple service contacts displayed larger disparities. For example, in 2006, inequalities were larger for DPT immunization (living standard CI = 0.0641; education CI = 0.0539), which requires three doses, than for BCG (living standard CI = 0.0164; education CI = 0.0121), which only requires one. Care-seeking for pneumonia displayed greater inequalities (poor/rich ratio of 0.70 in 2006) than care-seeking for diarrhea (0.89).

While using data from two different types of surveys enabled the study of trends over a 10-year period, it is also a source of some limitations of the study. Although DHS and MICS are similar in key respects and measure most of the same indicators for MNCH, and use the same methodology to calculate the wealth index, there are differences. In general, inequalities were found to be larger in the two MICS surveys compared to the two DHS surveys. For mortality this may be due to the fact that two different methods to estimate mortality had to be applied. The methods may suffer from different errors, for example random errors in sample surveys or systematic errors due to misreporting. However, we have not been able to reach a conclusion as to why MICS generally present larger inequalities for health and nutrition outcomes and intervention coverage. There were some differences in the definitions of the education, ethnicity and region variables. We explained in the methods section how we addressed these differences.

Another limitation of this study is that the measure of living standards - the wealth index - is correlated with other stratifying variables, such as education, ethnicity, and rural or urban place of residence, which raises another important point of discussion. We believe that differences in education, ethnicity, and residence have a direct impact of inequalities in health and nutrition outcomes and intervention coverage. However, one plausible alternative hypothesis is that the predominant source of inequality is living standard status and that the other stratifying variables are proxies for living standards. For example, inequality by ethnicity could be a reflection of the fact that ethnicity may proxy for being poor, less educated, and living in environments less conducive to good health outcomes.

Finally, in recent years there has been a growing recognition that there are potential problems with the CI as a tool to measure inequality [44]. For example, one study found that different rankings could be obtained if the measure of inequality is inversed, for example if inequalities in ill health are measured rather than inequalities in health (sometimes referred to as the “mirror problem”) [45]. This would be a particular problem if analysis of the development of the CI over time would show different trends depending on which of the two indicators would be used. We have tested our data for this possibility, and have not observed any differences in trends.

## Discussion

The findings of this study confirm Vietnam’s impressive progress in reducing child deaths; the mean IMR and U5MR decreased by more than 40% in 1997–2006. The findings also suggest that the distribution of RMNCH and nutrition outcomes and intervention coverage among different population groups is inequitable. Despite some progress, inequalities in health outcomes persisted between 1997 and 2006; the CI for IMR and U5MR became slightly more inequitable. Inequalities in coverage of health interventions decreased between 1997 and 2006, which due to time lags of effects of certain interventions, such as immunization, on health outcomes may result in reduced inequalities in infant and U5MR in subsequent surveys. Increasing inequalities in health outcomes despite decreasing inequalities in health service coverage may also suggest that other factors related to Vietnam’s rapid socioeconomic development, for example transportation and living environments, may drive inequalities in health outcomes.

Generally we found larger inequalities by living standards compared to inequalities by education of mother, ethnicity, region, urban versus rural residence, and sex of child. An implication of this finding is that government policies aimed at reducing inequalities - such as free health insurance cards for the poor, ethnic minorities and children under six - may benefit from a complimentary policy instrument that also increases income, such as conditional cash transfers. This policy instrument provides a cash payment to households conditional upon carrying out actions such as attending growth monitoring sessions, receiving immunizations and getting regular health check-ups [46].

Inequalities in child nutrition were larger for underweight and stunting, which signal long-term nutritional deficiencies, compared to wasting, which is usually a result of a sudden, short-term reduction in nutritional intake. Given that we only had data on these three outcomes for 2000, we were not able to study trends over time.

The determinants of birth weight are multi-factorial, but it is well known that malnutrition of the mother plays an important role, not just during pregnancy but in her whole life leading up to pregnancy. There was a large decrease in inequalities in low birth weight over time (the CI in 2006 was less than half of what it was in 1997), which suggests that the nutritional status of pregnant women in the poorer quintiles of the population is catching up with that of those in the richer quintiles, but it is not clear from this analysis why that may be the case.

Our study found that inequalities were greater for interventions that require more than one service contact, such as DPT immunization, which needs to be taken in three doses to be fully effective, and antenatal care, which should ideally be provided at least four times during pregnancy, or at least three times as recommended by the Ministry of Health of Vietnam [47]. This suggests that inequalities can be reduced by strengthening outreach by frontline workers such as community health workers and village health workers, particularly in rural and remote areas, to facilitate antenatal care visits for pregnant women, follow-up visits for mothers who have recently delivered, and health check-ups and growth monitoring sessions for children. It also suggests that addressing financial and other access barriers needs to complement targeted investments in the health system. Such demand-side barriers include distance to health facilities, transportation network, opportunity costs for the patients and care-takers, and cultural factors [48].

Inequalities were also larger for interventions that require support from the health system, such as skilled birth attendance, compared to interventions that can be delivered with less support from the health system through campaigns, such as certain immunizations and Vitamin A supplementations. A similar pattern was found by a World Bank study of inequalities in health in 56 countries [11], the Countdown to 2015 equity analyses [22,28,49] and a study of equity in MNCH in Thailand [50]. This suggests that policies aiming to reduce inequalities should invest in health system strengthening, particularly in areas that are disproportionately inhabited by the poor and other vulnerable groups, such as ethnic minorities. Current investment in Vietnam favors urban areas and higher levels of the health system, such as tertiary hospitals, at the expense of investments in primary care [51].

The two indicators related to breastfeeding displayed a pro-poor distribution of inequalities. Further study is required to shed light on the reasons behind this result, but possible reasons may include changing social norms and behaviors among the growing number of more affluent households, cultural beliefs, lack of means by the poor to seek alternative nutrition intake sources for their infants should they wish to do so (even if not desirable from a health point of view), and marketing of infant formula - often next to schools - in affluent urban areas of Vietnam.

## Conclusions

Three main policy implications emerge from this study. First, persistent inequalities suggest the need to address financial and other access barriers, for example by subsidizing health care for the poor and ethnic minorities and by support from other sectors, for example in strengthening transportation networks. This should be complemented by careful monitoring and evaluation of current program design and implementation to ensure effective and efficient use of resources. Second, greater inequalities for interventions that require multiple service contacts imply that inequalities could be reduced by strengthening information and service provision by community and village health workers to promote and sustain timely care-seeking. Finally, larger inequalities for interventions that require a fully functioning health system suggest that investments in health facilities and human resources, particularly in areas that are disproportionately inhabited by the poor and ethnic minorities, may contribute to reducing inequalities.

To shed further light on inequalities in RMNCH in Viet Nam, future research could benefit from a technique called “decomposing” the CI to analyze what specific factors drive its size (O’Donnell et al., [2008]). Another pertinent topic for further study is to explore how income inequalities changed in 1997–2006 and to what degree such changes were associated with changes in inequalities in RMNCH and nutrition outcomes and intervention coverage.

## Endnote

^a^The concentration curve displays the share of health accounted for by cumulative proportions of individuals in the population ranked from poorest to richest or from lowest to highest education level. The concentration curve plots the cumulative percentage of the health variable on the y-axis and the cumulative percentage of the population by living standards (or education) on the x-axis. If the distribution of a health outcome or intervention coverage is perfectly equal, the concentration curve will be a straight 45-degree line, sometime referred to as the line of equality.

## Abbreviations

BCG: Bacille Calmette Guerin (vaccination for tuberculosis); CI: Concentration index; DHS: Demographic and Health Survey; DPT: Diphtheria, pertussis and tetanus (vaccination); GNI: Gross national income; MDG: Millennium Development Goals; MICS: Multiple Indicator Cluster Survey; PMNCH: Partnership for Maternal, Newborn & Child Health; RMNCH: Reproductive, maternal, newborn and child health; WHO: World Health Organization.

## Competing interests

The authors declare that they have no competing interests.

## Authors’ contribution

All authors participated in the conceptualization and design of the study. HA carried out the data analysis. All authors participated in interpretation of the results and drafting of the manuscript. All authors have approved the submitted manuscript.

## Pre-publication history

The pre-publication history for this paper can be accessed here:

http://www.biomedcentral.com/1472-6963/12/456/prepub

## References

[B1] World Bank http://data.worldbank.org/country/vietnam. [Accessed on 29 May 2012]

[B2] UNICEF, WHO, World Bank, UN DESA/Population Division Levels and trends in child mortality: report 2012. Estimates developed by the UN Inter-agency Group for Child Mortality Estimation 2012 New York: UNICEF

[B3] WHO, UNICEF, UNFPA, World Bank Trends in maternal mortality: 1990–2010 2012 Geneva: World Health Organization

[B4] Knowles JC Bales S Cuong LQ Oanh TTM Luong DH Health equity in Viet Nam - A situational analysis focused on maternal and child mortality 2009 Hanoi: UNICEF

[B5] Hoa DP Nga NT Malqvist M Persson LA Persistent neonatal mortality despite improved under-five survival: a retrospective cohort study in northern Vietnam Acta Paediatr 2008 97 166 170 10.1111/j.1651-2227.2007.00604.x 18254906

[B6] Ekman B Liem NT Duc HA Axelson H Health insurance reform in Vietnam: a review of recent developments and future challenges Health Policy Plan 2008 23 252 263 10.1093/heapol/czn009 18424793

[B7] Axelson H Bales S Minh PD Ekman B Gerdtham U-G Health financing for the poor produces promising short-term effects on utilization and out-of-pocket expenditure: evidence from Vietnam Int J Equity Health 2009 8 20 10.1186/1475-9276-8-20 19473518 PMC2694203

[B8] Wagstaff A Estimating health insurance impacts under unobserved heterogeneity: the case of Vietnam's Health Care for the Poor Health Econ 2009 19 189 208 19248053 10.1002/hec.1466

[B9] Anwar I Sami M Akhtar N Chowdhury ME Salma U Rahman M Koblinsky M Inequity in maternal health-care services: evidence from home-based skilled-birth-attendant programmes in Bangladesh WHO Bulletin 2008 86 252 259 10.2471/BLT.07.042754 PMC2647426 18438513

[B10] Gakidou E Vayena E Use of modern contraception by the poor is falling behind PLoS Med 2007 2 e31 17284155 10.1371/journal.pmed.0040031 PMC1796626

[B11] Gwatkin DR Rutstein S Johnson K Suliman E Wagstaff A Amouzou A Socioeconomic differences in health, nutrition, and population within developing countries - an overview 2007 Washington, DC: World Bank 18293634

[B12] Houweling TAJ Kunst AE Socio-economic inequalities in childhood mortality in low- and middle-income countries: a review of the international evidence Br Med Bull 2010 93 7 26 10.1093/bmb/ldp048 20007188

[B13] Black RE Morris SS Bryce J Where and why are 10 million children dying every year? Lancet 2003 361 2226 2234 10.1016/S0140-6736(03)13779-8 12842379

[B14] Schellenberg JA Victora CG Mushi A de Savigny D Schellenberg D Mshinda H Bryce J Tanzania Integrated Management of Childhood Illness MCE Baseline Household Survey Study Group Inequities among the very poor: health care for children in rural southern Tanzania Lancet 2003 361 561 566 10.1016/S0140-6736(03)12515-9 12598141

[B15] Wang L Determinants of child mortality in LDCs: empirical findings from demographic and health surveys Health Policy 2003 65 277 299 10.1016/S0168-8510(03)00039-3 12941495

[B16] Wamani H Tylleskar T Nordrehaug Astrom A Tumwine JK Peterson S Mothers' education but not fathers' education, household assets or land ownership is the best predictor of child health inequalities in rural Uganda Int J Equity Health 2004 3 9 10.1186/1475-9276-3-9 15482596 PMC529301

[B17] Victora CG Matijasevich A Silveira MF Santos I Barros AJ Barros FC Socio-economic and ethnic group inequities in antenatal care quality in the public and private sector in Brazil Health Policy Plan 2010 25 253 261 10.1093/heapol/czp065 20123940 PMC2889278

[B18] Fotso JC Child health inequities in developing countries: differences across urban and rural areas Int J Equity Health 2006 5 9 10.1186/1475-9276-5-9 16831231 PMC1544325

[B19] Victora CG Wagstaff A Armstrong Schellenberg J Gwatkin D Claeson M Habicht J-P Applying an equity lens to child health and mortality: more of the same is not enough Lancet 2003 362 233 241 10.1016/S0140-6736(03)13917-7 12885488

[B20] Measure DHS Demographic and Health Surveys Overviewhttp://www.measuredhs.com/aboutsurveys/dhs/start.cfm [Accessed on 29 May 2012]

[B21] UNICEF Multiple Indicator Cluster Surveyshttp://www.childinfo.org/mics.html [Accessed on 29 May 2012]

[B22] Barros AJD Ronsmans C Axelson H Loaiza E Bertoldi AD França GVA Bryce J Boerma JT Victora CG Equity in maternal, newborn, and child health interventions in Countdown to 2015: a retrospective review of survey data from 54 countries Lancet 2012 379 1225 1233 10.1016/S0140-6736(12)60113-5 22464386

[B23] Boerma JT Bryce J Kinfu Y Axelson H Victora CG Mind the gap: equity and trends in coverage of maternal, newborn and child health services in 54 Countdown countries Lancet 2008 371 1259 1267 18406860 10.1016/S0140-6736(08)60560-7

[B24] Abdesslam B Uwe H Social inequalities, regional disparities and health inequity in North African countries Int J Equity Health 2011 10 23 23 10.1186/1475-9276-10-23 21627818 PMC3120653

[B25] Lloyd CB Hewett P Educational inequalities in the midst of persistent poverty: diversity across Africa in educational outcomes Journal of International Development 2009 21 1137 1151 10.1002/jid.1650

[B26] Deaton A The analysis of household surveys: a microeconometric approach to development policy 1997 Baltimore, MD: Johns Hopkins University Press

[B27] Hill K Pande R Mahy M Jones G Trends in child mortality in the developing world: 1960–1996 1999 New York: UNICEF

[B28] United Nations Indirect Techniques for Demographic Estimation 1983 New York: United Nations

[B29] United Nations QFIVE – United Nations program for child mortality estimation 1990 New York: United Nations

[B30] Black RE Cousens S Johnson HL Lawn JE Rudan I Bassani DG Jha P Campbell H Fischer Walker C Cibulskis R Eisele T Liu L Mathers C for the Child Health Epidemiology Reference Group of WHO and UNICEF Global, regional and national causes of child deaths Lancet 2010 375 1969 1987 10.1016/S0140-6736(10)60549-1 20466419

[B31] Black RE Allen LH Bhutta ZA Caulfield LE de Onis M Ezzati M Mathers C Rivera J for the Maternal and Child Undernutrition Study Group Maternal and child undernutrition: global and regional exposures and health consequences Lancet 2008 371 243 260 10.1016/S0140-6736(07)61690-0 18207566

[B32] Blanc AK Wardlaw T Monitoring low birth weight: an evaluation of international estimates and an updated estimation procedure WHO Bulletin 2005 83 178 185 PMC2624216 15798841

[B33] Bhutta Z Chopra M Axelson H Berman P Boerma T Bryce J Bustreo F Cavagnero E Cometto G Daelmans B de Francisco A Fogstad H Gupta N Laski L Lawn J Maliqi B Mason E Pitt C Jennifer Requejo J Starrs A Victora CG Wardlaw T Countdown to 2015 decade report (2000–10): taking stock of maternal, newborn, and child survival Lancet 2010 375 2032 2044 10.1016/S0140-6736(10)60678-2 20569843

[B34] The Partnership for Maternal, Newborn Child Health Essential interventions, commodities and guidelines for reproductive, maternal, newborn and child health: a global review of the key interventions related to reproductive, maternal, newborn and child health (RMNCH) 2011 Geneva: PMNCH

[B35] Jones G Steketee RW Black RE Bhutta ZA Morris SS Bellagio Child Survival Study Group How many child deaths can we prevent this year? Lancet 2003 362 65 71 10.1016/S0140-6736(03)13811-1 12853204

[B36] Campbell OMR Graham WJ on behalf of The Lancet Maternal Survival Series steering group Strategies for reducing maternal mortality: getting on with what works Lancet 2006 368 1284 1299 10.1016/S0140-6736(06)69381-1 17027735

[B37] Filmer D Pritchett L Estimating wealth effects without expenditure data - or tears: an application to educational enrollments in states of India Demography 2001 38 115 133 11227840 10.1353/dem.2001.0003

[B38] Rutstein SO Johnson K The DHS Wealth Index 2004 Calverton, MD: ORC Macro

[B39] Dang HA Hall G, Patrinos H Vietnam: A widening poverty gap for ethnic minorities Indigenous Peoples, Poverty and Development 2010 World Bank, Washington, DC 1 35

[B40] O’Donnell O van Doorslaer E Wagstaff A Lindelow M Analyzing health equity using household survey data: a guide to techniques and their implementation 2008 Washington, DC: World Bank

[B41] Kakwani NC Wagstaff A van Doorslaer E Socioeconomic inequalities in health: measurement, computation and statistical inference J Econom 1997 77 87 104 10.1016/S0304-4076(96)01807-6

[B42] Wagstaff A Socioeconomic inequalities in child mortality: comparisons across nine developing countries WHO Bulletin 2000 78 19 29 PMC2560599 10686730

[B43] Wagstaff A Bryce J Bustreo F Claeson M WHO-World Bank Child Health and Poverty Working Group Child health: reaching the poor Am J Public Health 2004 94 726 736 10.2105/AJPH.94.5.726 15117689 PMC1448326

[B44] Erreygers G Correcting the concentration index J Health Econ 2009 28 504 515 10.1016/j.jhealeco.2008.02.003 18367273

[B45] Clarke PM Gerdtham U-G Johannesson M Bingefors K Smith L On the measurement of relative and absolute income-related health inequality Soc Sci Med 2002 55 1923 1928 10.1016/S0277-9536(01)00321-5 12406461

[B46] Lagarde M Haines A Palmer N Conditional cash transfers for improving uptake of health interventions in low- and middle-income countries: a systematic review JAMA 2007 298 1900 1910 10.1001/jama.298.16.1900 17954541

[B47] Ministry of Health National Standard Guidelines on Reproductive Health Services 2003 Hanoi: Ministry of Health

[B48] Ensor T Cooper S Overcoming barriers to health service access: influencing the demand side Health Policy Plan 2004 19 69 79 10.1093/heapol/czh009 14982885

[B49] Victora CG Barros AJD Axelson H Bhutta ZA Chopra M França GVA Kerber K Kirkwood B Newby H Ronsmans C Boerma T Equity in coverage of maternal and child health interventions in 35 Countdown to 2015 countries: an analysis of national surveys Lancet 2012 380 1149 1156 10.1016/S0140-6736(12)61427-5 22999433

[B50] Limwattananon S Tangcharoensathien V Prakongsai P Equity in maternal and child health in Thailand WHO Bulletin 2010 88 420 427 10.2471/BLT.09.068791 PMC2878146 20539855

[B51] Lieberman SS Wagstaff A Health financing and delivery in Vietnam: looking forward 2009 Washington, DC: World Bank

